# The left–right side-specific endocrine signaling in the effects of brain lesions: questioning of the neurological dogma

**DOI:** 10.1007/s00018-022-04576-9

**Published:** 2022-10-11

**Authors:** Georgy Bakalkin

**Affiliations:** Department of Pharmaceutical Biosciences, Uppsala University, Box 591, SE-751 24 Uppsala, Sweden

**Keywords:** Traumatic brain injury, Stroke, Cross association, Contralateral effects, Neuroendocrine system, Neurohormones, Opioid peptides, Left-right asymmetry, Pituitary, Hypothalamus, Cerebral palsy

## Abstract

Each cerebral hemisphere is functionally connected to the contralateral side of the body through the decussating neural tracts. The crossed neural pathways set a basis for contralateral effects of brain injury such hemiparesis and hemiplegia as it has been already noted by Hippocrates. Recent studies demonstrated that, in addition to neural mechanisms, the contralateral effects of brain lesions are mediated through the humoral pathway by neurohormones that produce either the left or right side-specific effects. The side-specific humoral signaling defines whether the left or right limbs are affected after a unilateral brain injury. The hormonal signals are released by the pituitary gland and may operate through their receptors that are lateralized in the spinal cord and involved in the side-specific control of symmetric neurocircuits innervating the left and right limbs. Identification of features and a proportion of neurological deficits transmitted by neurohormonal signals vs. those mediated by neural pathways is essential for better understanding of mechanisms of brain trauma and stroke and development of new therapies. In a biological context, the left–right side-specific neuroendocrine signaling may be fundamental for the control of the left- and right-sided processes in bilaterally symmetric animals.

## Introduction: the neural and novel neuroendocrine “cross association” concepts

**Fig. 1:**
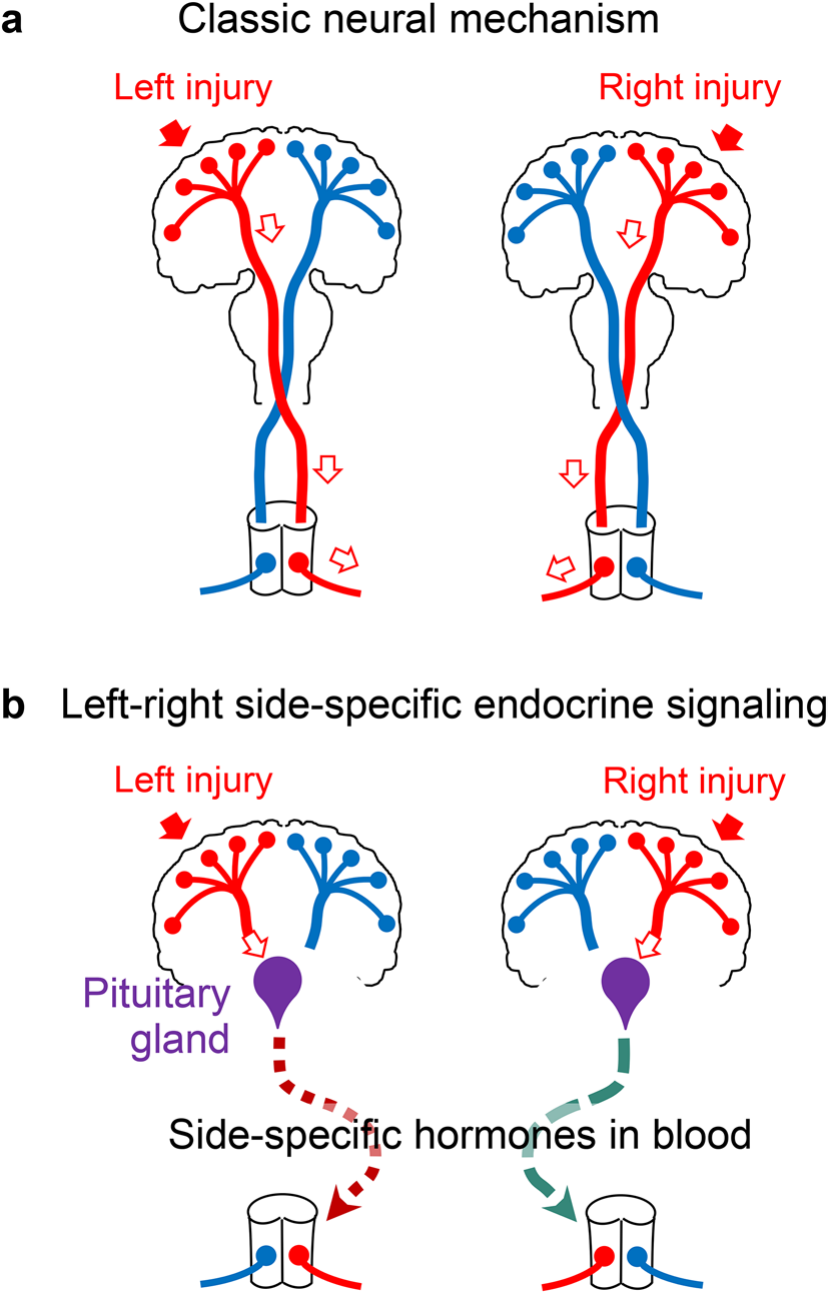
The classic neural and the novel left–right side-specific neuroendocrine mechanisms of signaling from injured brain to the contralateral body side. **a** In the classic mechanism, a unilateral brain injury impairs the descending neural tracts that decussate and control contralateral hindlimbs. **b** In the endocrine mechanism, brain injury stimulates the release of side-specific neurohormones from the pituitary gland into the blood, that then bind to neuronal receptors that are lateralized in the spinal cord and/or afferent neurons, and induce contralateral sensorimotor deficits [15]. The neurohormonal signaling operates in addition to the neural mechanism, and contributes to enduring side-specific neuroplastic changes in the spinal cord that underlie asymmetric postural and motor deficits such as hemiparesis and hemiplegia [15]

Contralateral effects of head injuries were already noted by Hippocrates (460–380 BC) [1–4]. This neurologic conundrum was explained 500 years later by Aretaeus of Cappadocia who emphasized that “the cause of this is the interchange in the origins of the nerves... each of them passes over to the other side from that of its origin, decussating each other in the form of the letter X” [4, 5]. In 1709–1710, Pourfour du Petit and Mistichelli [6, 7] identified the pyramids in the lower medulla as the site of the motor tract decussation, and then, in 1810, Gall and Spürzheim coined the term *décussation des pyramides* [4, 8]. These works laid the basis for the central neurology concept known as *cross association* [1, 4]. The concept states that each cerebral hemisphere is functionally connected to the contralateral side of the body through the decussating neural tracts (Fig. 1a). Since that time a cause of the contralateral effects of brain lesions has been considered as solely neuroanatomical—based on the decussation of the descending neural pathways [5, 9].

This review focuses on a novel phenomenon—the left–right side-specific neuroendocrine signaling that mediates the contralateral effects of brain lesions, in addition to the descending neural tracts (Fig. 1b). Early animal studies by Anna DiGiorgio [10] and subsequent works by others [11–14] demonstrated that a unilateral injury of the cerebrum or cerebellum causes hindlimb postural asymmetry (HL-PA), a proxy for neurological deficits with flexion on the contralesional or ipsilesional side, respectively. The asymmetry persisted after complete spinal transection implying that the effects of brain lesions are encoded by plastic rearrangements in spinal neurocircuitries or “pathological spinal memory”. The conceptual shift in understanding the brain injury-induced neurological deficits was triggered by experiments in which the descending neural tracts were disabled by complete transection of the spinal cord before the brain was injured [15]. Strikingly, rats with transected spinal cord and a unilateral injury of the hindlimb sensorimotor cortex did develop HL-PA with contralateral flexion along with asymmetry in hindlimb withdrawal reflexes and asymmetric gene expression patterns in lumbar spinal cord. The left or right hindlimb flexion was induced by the right- and left-side brain injury, respectively. These experiments uncovered the existence of an extraspinal pathway that bypasses descending neural tracts and conveys information on brain injury and its side to the lumbar spinal neurocircuits [15] (Fig. 1b). The brain injury-induced postural effects were abolished by hypophysectomy and were mimicked in animals with intact brain by transfusion of serum from animals with brain injury. Two humoral factors that mediate the effects of left brain injury were identified as Arg-vasopressin and β-endorphin. These neurohormones are produced in the hypothalamus and pituitary gland, and by themselves evoke asymmetric motor responses with the right hindlimb flexion in rats with intact brain [15]. The discovered neuroendocrine “cross association” signaling may trigger and tonically control enduring spinal plasticity that underlies lasting neurological deficits such as hemiparesis and hemiplegia. In a broader biological context, this signaling could be a part of the fundamental mechanism that selectively regulates the left and right body sides in bilaterians.

Here, we first highlight clinical and experimental findings that led to the discovery of the left–right side-specific neuroendocrine phenomenon. Starting from the contralesional effects of traumatic brain injury (TBI) and stroke in clinical settings and experimental animal models, we then detail the HL-PA model with lateralized spinal plasticity, and emphasize the unusual left–right side-specific actions of neurohormones as the messengers in the side-specific humoral signaling. Contributions of the neurohormonal signaling to lateralized neurological deficits secondary to TBI and stroke are then addressed. A role of the endocrine system in TBI and stroke, inferring that impairments of the side-specific humoral signaling is an essential part of their pathophysiology, is outlined. Finally, a contribution of the left–right side-specific neuroendocrine signaling to intergenerational transmission of neurological signs is discussed (see also our “Perspective” article [16]).

## Contralateral effects of TBI and stroke

TBI and stroke damage multiple brain regions leading to postural and sensorimotor impairments [17–26]. The motor deficits include motor weakness, loss of voluntary movements and dexterity, spasticity, asymmetric limb reflexes and abnormal posture that typically develop on the contralesional side. These impairments contribute to dynamic control asymmetry with spared ipsilesional limbs, weight-bearing asymmetry and impaired body sway control. The postural and motor impairments are often defined as a loss of symmetry in body posture and limb functions, while the regained symmetry is a characteristic of functional recovery [27, 28]. Among the consequences of stroke and TBI, impairments in posture have the largest impact on activities of daily living. The current view is that these effects are mediated by neural pathways that descend from the cerebral cortex to motoneurons in the brain stem and spinal cord [20–26].

Aberrant activity of the descending neural tracts and the evolved pathological spinal plasticity contribute to the asymmetric deficits [23, 25, 26, 29–35]. The impaired signaling through the corticospinal and rubrospinal tracts results in spasticity and deficits in voluntary and skilled movements. Abnormal reflexes and posture may be caused by an aberrant activity of the reticulospinal and vestibulospinal tracts, as well as deafferentation-induced spinal plasticity [25, 26, 36].

Multiple contralateral changes in monosynaptic and polysynaptic reflexes after TBI and stroke were identified in clinical studies [37–43]. Changes in the polysynaptic nociceptive withdrawal reflexes were considered as electrophysiological biomarkers for reorganization of spinal circuitry converging sensory input and descending motor commands [44–52]. The withdrawal reflexes are commonly affected in hemiparetic patients after stroke [52–55]. Their kinematic responses were increased while their modulation was impaired in patients with hemiparesis [55, 56]. The withdrawal reflexes-based electrical therapy facilitated the processes of gait rehabilitation in hemiparetic patients by improving both walking velocity and gait symmetry [54, 57].

Contralateral neurological deficits often develop differently after injury to the left and right hemisphere. The right-side stroke leads to poorer postural responses in quiet and perturbed balance compared to injury of the left hemisphere implying a more prominent role of the right hemisphere in efferent control of balance [58]. The contraversive pushing called “Pusher syndrome”, a serious postural impairment, is more common in patients with lesions of the right hemisphere [59]. Impairments of a trunk control often depend on the lesion side. “Apraxic responses” are more frequent after the left hemisphere injuries, whereas “postural instability” prevail after lesions of the right hemisphere [60]. Pathophysiological mechanisms of neurological deficits along with the differences between lesions of left and right hemisphere are not well understood.

## Hindlimb postural asymmetry (HL-PA) induced by unilateral brain lesions

**Fig. 2:**
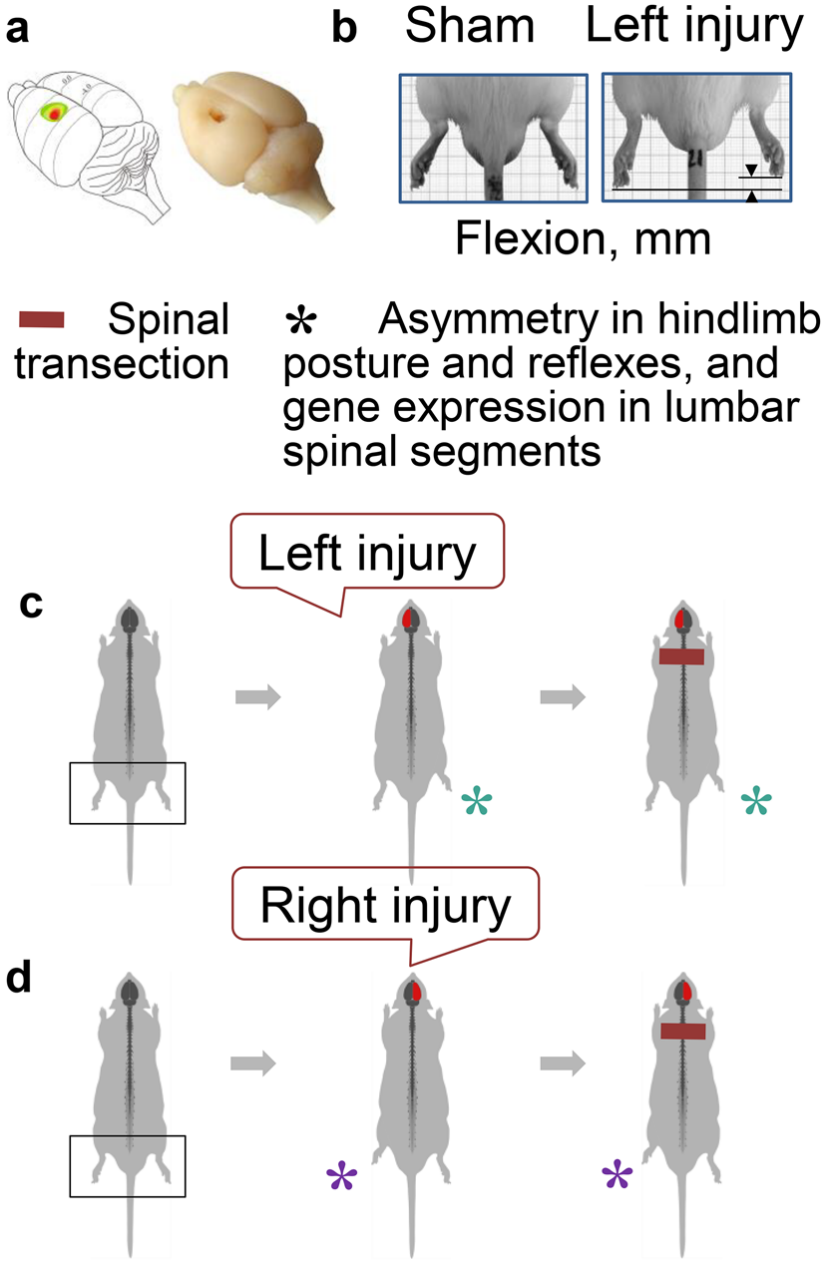
Enduring spinal plasticity underlying formation of asymmetric hindlimb posture after a unilateral brain injury. Persistence of the asymmetry after complete transection of the thoracic spinal cord. **a** Representative unilateral brain ablation injury of the right hindlimb representation area (modified from [15]). **b** Analysis of hindlimb postural asymmetry (HL-PA), a proxy for neurological deficits. HL-PA, asymmetry in withdrawal reflexes analyzed by recording EMG of flexors and extensors of the left and right hindlimbs, and expression of neuroplasticity-related and neuropeptide genes in the lumbar spinal cord were studied as readouts of the effects of unilateral brain lesions [13, 14, 70]. **c**, **d** HL-PA with right flexion was induced by the left-sided brain injury, while HL-PA with left flexion was induced by right-sided brain injury [13, 14, 70]. A thoracic spinal transection does not reverse the expression of HL-PA. The asterisks denote the side of flexed limb

Changes in functions of the contralesional limbs can be induced by aberrant activity of the motor cortex, direct and indirect descending pathways and the spinal circuits that they target [25, 61–63]. Early animal studies showed that unilateral brain lesions cause asymmetric changes in posture and reflexes and that these changes persist after complete spinal cord transection [10, 11]. In that studies, the ipsilateral hindlimb was flexed after a hemicerebellar lesion, and the effect was retained after transection of the spinal cord. This phenomenon was regarded as pathological spinal memory. Other studies demonstrated that the lateral spinal cord hemisection enhanced ipsilateral monosynaptic and polysynaptic reflexes, and these effects were sustained after caudal spinal cord transection [64–69]. In accordance with the neural “cross association” concept, both a unilateral focal ablation injury of the hindlimb sensorimotor cortex and the controlled cortical TBI produced asymmetry in hindlimb posture with flexion of the contralesional hindlimb, contra-ipsilesional differences in musculo-articular resistance of hindlimbs to stretch, and the contralesional activation of the nociceptive hindlimb withdrawal reflexes, all of which persisted after decerebration and spinal cord transection [13–15, 70] (Fig. 2). These contralateral effects are the focus of this review; therefore, we will discuss the resulting HL-PA in more details.

The HL-PA induced by unilateral brain lesions is a proxy for postural impairments and represents a model of “hemiplegic posture” [10, 11, 13–15, 70]. HL-PA was observed in animals under anesthesia and in unanesthetized animals that were decerebrate after brain injury. HL-PA remained stable after complete spinal cord transection [10, 11, 13–15, 70]. Withdrawal and stretch reflexes do not likely contribute to HL-PA because they are abolished immediately and for days after complete spinal cord transection and drastically decreased under anesthesia (discussed in details in [13]). This is also supported by the observation that bilateral deafferentation of the lumbar spinal cord segments from L1 to S2 does not affect HL-PA induced by the right-side UBI. However, the asymmetric postural responses were eliminated by bilateral lumbar dorsal rhizotomy after the left-side brain injury. Thus two mechanisms, one dependent on and one independent of afferent input could account for asymmetric hindlimb responses after the left and right side brain injuries, respectively. The later mechanism could be based on the brain injury-evoked tonic activity of spinal motoneurons. On the other hand, segmental reflexes mediated by proprioceptive neurons that are activated by group II muscle afferents, remain functional and maintain muscle tone after spinal cord transection [71–73], and could contribute to HL-PA after the left side injury. A role of these mechanisms remains to be established.

The HL-PA animal model is well reproducible and quantifiable. It is technically simple and allows researchers to address a variety of experimental questions in a limited time frame [12–15, 70, 74]. The HL-PA model permits analysis of extraspinal neurophysiological mechanisms of motor deficits because the asymmetry persists after complete transection of the descending neural tracts [13, 14, 70]. The postural and reflex responses are directed along the left–right axis and, therefore, could be used to study contra-ipsilesional and left–right side-specific processes.

The HL-PA model recapitulates several pathophysiological characteristics of the human upper motor neuron syndrome such as the “hemiplegic posture”. In this model, a unilateral ablation brain injury (UBI) produces contralateral effects. In animals, formation of HL-PA with contralesional flexion parallels with motor impairments of the contralateral hindlimb that are exhibited in locomotor tasks [15]. In rats with right-side brain injury, the postural effects depend on the efferent but not on afferent input as evident from persistence of HL-PA after bilateral lumbar deafferentation [13]. This feature of HL-PA resembles “spastic dystonia” defined as “stretch- and effort-unrelated sustained involuntary muscle activity following central motor lesions” in patients [75, 76]. The translational value of this model is exemplified by formation of HL-PA with the contralesional hindlimb flexion after the controlled cortical impact, a clinically relevant TBI model [14].

## Brain injury-induced lateralized spinal plasticity

Persistence of asymmetry in posture and reflexes after spinal cord transection (Fig. 2c, d) suggests that the brain injury-induced neurological deficits are encoded in the spinal cord [10, 11, 13, 14, 69, 70, 77]. Spinal neural circuits might be rewired and spinal molecular systems regulating these processes activated. Although spinal molecular systems mediating the effects of brain injury on neurological deficits have not yet been identified, several studies described cellular and molecular alterations in the cervical and lumbar spinal cords after brain injuries [78–80].

A distinct period of structural plasticity, growth factor expression, and inflammatory cytokine production after stroke was identified in the cervical spinal cord [13, 78, 79]. Stroke results in upregulation of growth-associated protein-43, brain-derived neurotrophic factor and neurotrophin-3. A period of heightened plasticity was associated with elevated levels of inflammatory cytokines tumor necrosis factor-alpha and interleukin-6. Neuroplastic responses peaked during the first 2 weeks after stroke and then sharply declined. Spinal plasticity correlated with the severity of cortical injury and temporally matched periods of accelerated spontaneous recovery of skilled reaching function.

Consistently, a focal controlled cortical impact TBI produced changes in molecular systems regulating neuronal plasticity in the lumbar spinal cord [80]. TBI alters expression of the *Tgfb1*, *c-Fos*, *Bdnf*, and *Gap43* neuroplasticity genes. The *Tgfb1* mRNA levels and the number of c-Fos-positive cells were significantly elevated in the TBI vs. sham-injured rats [80]. Expression patterns were markedly asymmetrical with higher levels on the contralesional side. The TBI-induced molecular alterations in the lumbar spinal cord could underlie a rearrangement of locomotor spinal neural circuits and contribute to either maladaptive motor responses or motor recovery.

Two patterns of the UBI-induced molecular changes in the spinal cord were envisaged. In the contralateral pattern, molecular changes differ between contralesional and ipsilesional sides, and these differences are similar for the left- and right-sided injury. In the left–right pattern, the contra-ipsilesional differences in the spinal circuits depend on the side of brain injury. To distinguish these patterns, the effects of injury of the left and right hemisphere on gene expression in the left and right lumbar spinal cord were compared [13]. Expression of several neuroplasticity-related genes was altered including *Grin2a* and *Tgfb1* that were down- and upregulated, respectively. The *Grin2a* gene encodes a subunit of the glutamate receptors that regulates neural plasticity. *Tgfb1* gives rise to Transforming Growth Factor β1 regulating inflammation, expression of neuropeptides, and glutamate neurotoxicity. These changes were similar after the left and right side brain injuries whereas regulatory networks of the neuroplasticity-related genes were differentially affected. The left-side brain injury strongly reduced coordination of gene expression between the left and right halves of the lumbar spinal cord, and within each half. The effects of the right side injury were much less pronounced [13].

In the neural “cross association” concept, the contralateral spinal plasticity is caused by direct neural influences descending from injured brain and underlies the persistence of HL-PA and asymmetry in reflexes. In parallel with this mechanism, an abnormal spinal plasticity could be enabled through an extraspinal route by the contra-ipsi or left–right side-specific humoral signaling that will be discussed in the next sections.

## Left–right side-specific effects of neuropeptides and spinal asymmetry

Neuropeptides including opioid peptides are neuromodulators and paracrine regulators. They exert specific and coherent control over formation and rewiring of neural circuits that regulate behavior, endocrine system and sensorimotor functions [81, 82]. Opioid peptides and receptors are expressed in the dorsal and ventral spinal cord, where they regulate processing of sensory information, reflexes and motor functions [83–88]. Opioid receptors of δ- and µ-subtypes are expressed in the ventral horn neurons and have a role in motor control [83]. Opioids modify ventral root reflexes by inhibition of afferent signaling presynaptically, by postsynaptic inhibition of interneurons in the dorsal horn, and by actions on interneurons regulating activity of motoneurons in the ventral horn [83]. This may suppress the ipsilateral reflexes [87]. Targeting of opioid receptors in neurons that surround the central canal [83, 89] inhibits the spinal commissural pathways [90, 91] and contralateral reflexes [92]. The opioid and other neuropeptide systems as regulators of neural circuits could contribute an aberrant spinal plasticity triggered by signals from the injured brain.

### Induction of HL-PA by opioid peptides

**Fig. 3:**
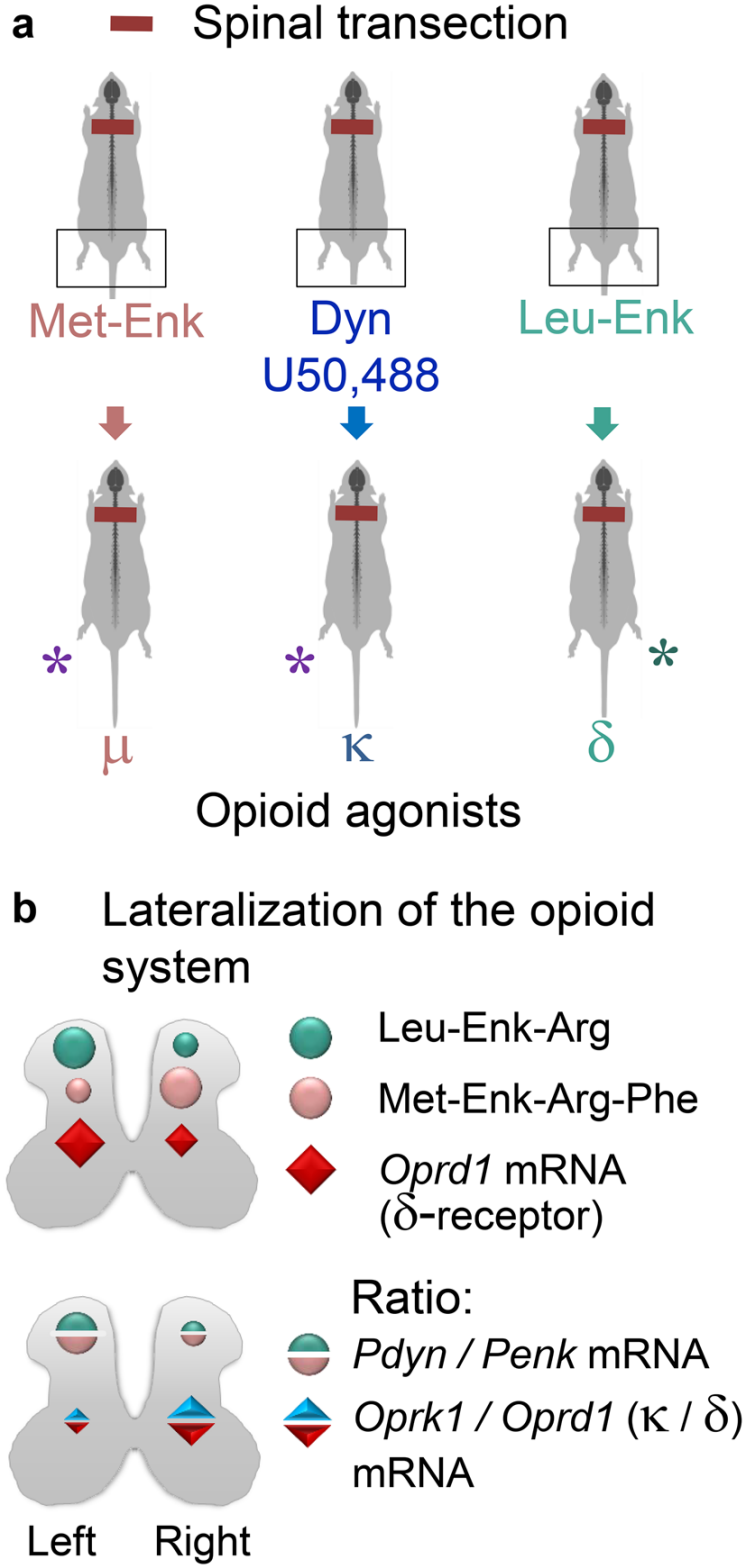
Left–right side-specific effects of opioid peptides on the HL-PA formation, and the underlying lateralization of the opioid system in the lumbar spinal cord. **a** Development of HL-PA with left hindlimb flexion was induced by Met-enkephalin (Met-Enk), the preferential endogenous µ-agonist, and by κ-agonists dynorphin (Dyn) and U50,488H in rats with thoracic spinal transection and no brain injury [14, 93, 94, 96]. HL-PA with right hindlimb flexion was induced by Leu-enkephalin (Leu-Enk), the preferential δ-agonist. **b** Lateralization of the opioid system in the lumbar spinal cord. Upper panel: Leu-enkephalin–Arg, the prodynorphin (Pdyn) marker, and the δ-opioid receptor (*Oprd1*) mRNA are lateralized to the left, whereas Met-enkephalin–Arg–Phe, the proenkephalin (Penk) marker, to the right [70]. Lower panel: left–right differences in the ratio of *Pdyn* / *Penk* mRNA levels and the ratio of κ- / δ-opioid receptor (*Oprk1*/*Oprd1*) mRNA levels. The relative left–right distribution of the prodynorphin and proenkephalin transcripts, and of their peptide products Leu-enkephalin–Arg and Met-enkephalin–Arg–Phe, exhibit similar lateralization patterns. The findings suggest that the left and right side-specific effects of opioid agonists are mediated through the lateralized opioid receptors. These lateralization patterns are similar with those in the cervical spinal cord in direction and extend [84]

An analysis of the spinal effects of the opioid peptides as regulators of spinal plasticity led to unanticipated findings: animals that were exposed to opioid peptides or synthetic opioids, exhibited different responses on their left and right sides that were manifested as formation of HL-PA [14, 70, 93–96] (Fig. 3a). More surprisingly, the flexion side was not random but was either on the left or right, and the sidedness was determined by the type of the compound administered. The left hindlimb was flexed after administration of the µ-/δ-agonist Met-enkephalin, and the selective κ-agonists dynorphin, bremazocine and U-50,488H (trans-3,4-Dichloro-N-methyl-N-[2-(1-pyrrolidinyl)cyclohexyl]-benzeneacetamide) [14, 93, 94, 96]. Conversely, Leu-enkephalin, which acts through the δ-receptor, caused the right hindlimb to flex [94, 96]. The HL-PA was robust, exhibited by most animals after administration of opioid ligands, and blocked by the nonselective opioid antagonist naloxone. The effects were produced after intrathecal or intravenous administration of the compounds, and, therefore, a preferential targeting of either the left or right side of the body by the opioids as a cause of this asymmetry was ruled out. The HL-PA was induced in animals with transected spinal cord, while in naïve animals the expression of the left or right side-specific effects is probably prevented by the descending compensatory influences.

Previously reported functional and molecular asymmetries are described as quantitative with mostly minor, about 30% differences between the left and right sides of the brain or spinal cord. The HL-PA experiments demonstrated that opioid peptides and synthetic opioids evoke responses either on the left or right side [14, 70, 93–96] (Fig. 3a). This is apparently the first example of a qualitative directional asymmetry in the central nervous system. These findings suggest that the left and right-sided processes are differentially regulated by neuropeptides. Specifically, mirror-symmetric neurocircuits that project to the left and right hindlimb muscles may be differentially controlled by the endogenous opioid peptides acting through µ-, δ- or κ-opioid receptors.

### Asymmetric organization of spinal neural circuits

Several studies uncovered asymmetry in functions and molecular organization of neural circuits in the spinal cord [13, 64–66, 84, 93, 94, 96–103]. Decussation of the descending neural fibres in human brain is asymmetrical [99]. More extensive and rostral crossing was noted for neural tracts projecting from the left hemisphere compared to those from the right hemisphere. This was associated with the larger size of the right vs. left spinal cord. Mono- and polysynaptic reflexes that were evoked by stimulation of the dorsal roots and recorded in the ventral roots displayed higher activity on the right side in intact rats and cats [64–66]. Similarly, the nociceptive hindlimb withdrawal reflexes evoked by electrical hindlimb stimulation and recorded by EMG exhibited higher activity on the right side in rats [13].

As described in the previous section, the opioid peptides induce asymmetric hindlimb responses with the affected side that is determined by the opioid administered. At the molecular level, there responses could be mediated through the opioid receptors that are lateralized in the spinal cord. Indeed, the opioid receptor and peptide genes display asymmetric expression [70, 84]. In the cervical spinal cord all three opioid receptors are lateralized to the left [84]. At the same time their proportions differ between the left and right ventral horns; the κ-receptor predominates on the right vs. left side. Analysis of gene regulatory networks demonstrated that expression patterns of the opioid genes are coordinated between the dorsal and ventral horns, while this coordination differed between the left and right sides. In the lumbar spinal cord the lateralization patterns were generally the same [70] (Fig. 3b). Expression of the δ-receptors was lateralized to the left whereas the proportion of κ- to δ-receptors (the *Oprk1*/*Oprd1* expression ratio) was higher on the right side. Opioid peptides were also lateralized with Leu-enkephalin-Arg (a prodynorphin marker) to the left, and Met-enkephalin-Arg-Phe (a proenkephalin marker) to the right. The ratio of prodynorphin to proenkephalin mRNA, and the ratio of Leu-enkephalin-Arg to Met-enkephalin-Arg-Phe peptides that are translated from these mRNAs, respectively, were substantially higher in the left half vs. right half of the lumbar spinal cord (Fig. 3b). Lateralization of the opioid system in the spinal cord could be a molecular basis for the left–right side-specific effects of opioid agonists. These findings also suggest that the opioid mechanisms in the left and right spinal cord are differentially involved in the lateralized processing of pain [103] and spinal neuroplastic responses to unilateral brain lesions.

Analysis of expression patterns of the opioid genes in the lumbar spinal cord in rats with spinal nerve ligation (i.e., a neuropathic pain model) supports this notion. Alterations in gene expression depend on neuropathy side and differ between the left and right spinal cord [103]. Changes in expression of μ-opioid receptor and proenkephalin genes differed between rats with the left and right side injury, while prodynorphin expression was similarly affected by left and right-side nerve damage. Notably, after the right-sided injury expression of the prodynorphin gene in the right ventral horn correlated negatively with withdrawal response thresholds, indicators of mechanical allodynia. However, after ligation of the left nerve the correlation between prodynorphin in the left ventral horn and the thresholds was positive. These findings implicate that dynorphins in the left and right spinal cord could mediate the effects of ipsilateral tactile stimulation in opposite directions, namely, through inhibition of the left vs. facilitation of the right hindlimb motor reflexes.

Lateralization bias in the opioid system raises the question as to whether such a pattern is general and exhibited by other neurotransmitters and neuropeptides. Analysis of the glutamate receptor genes revealed that the *Grin2b* gene coding for the NMDA receptor subunit, that is involved in circuit formation and synaptic plasticity, is expressed at higher levels in the left compared to the right lumbar spinal cord [13, 70]. Expression of this gene is also lateralized in the hippocampus that represents an essential part of structural and functional left–right asymmetry in this region [104, 105].

Another example of lateralized expression is the renin–angiotensin system that is involved in neuroprotection and pathological neuroplasticity in the central nervous system [74]. Analysis of this system in the left and right lumbar spinal cord of intact rats and of animals with a unilateral cortical injury of the left or right hemisphere demonstrated asymmetric expression of the *Ace*, *Agtr2* and *Ren* genes with higher levels on the left side. These genes code for angiotensin-converting enzyme, angiotensin receptor AT2 and renin, respectively. Coordination of the renin–angiotensin gene expression was asymmetrical, with stronger pattern on the right side, while the cortical injury induced shift to negative regulatory interactions between the renin–angiotensin genes and neuroplasticity-related genes in the contralateral spinal cord. Thus, expression of the opioid, glutamate and renin–angiotensin genes was lateralized, suggesting a role of these systems in a side-specific regulation of spinal neural circuits.

## Left–right side-specific opioid control of contralateral responses to brain injury

**Fig. 4:**
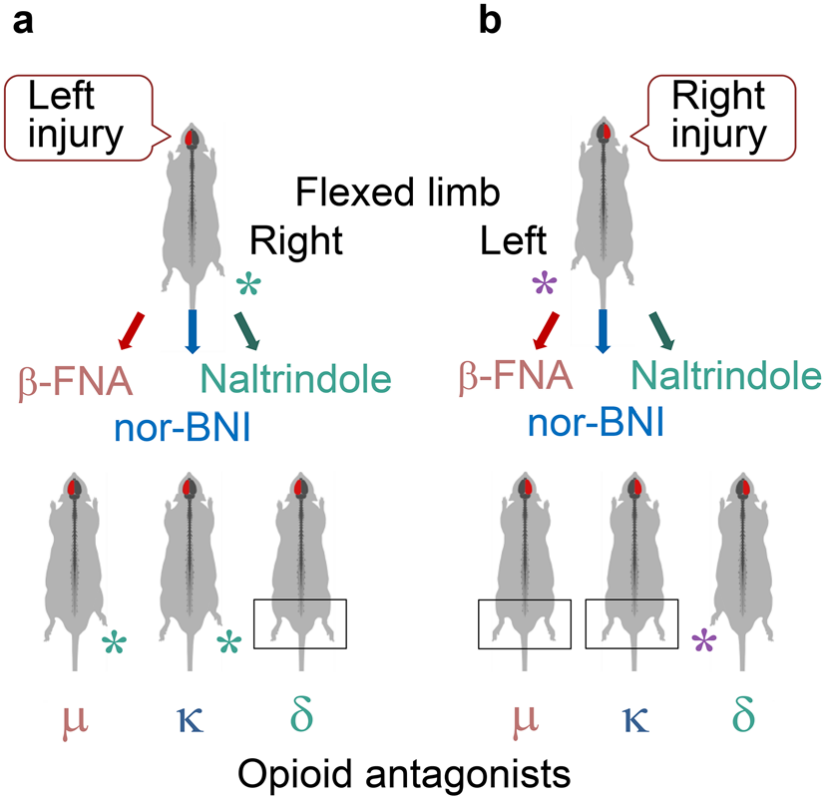
The effects of selective opioid antagonists on HL-PA formation depend on the side of brain injury. **a** β-Funaltrexamine (β-FNA) and nor-binaltorphimine (nor-BNI), the selective µ- and κ-opioid antagonists, respectively, block the HL-PA induced by the right-sided ablation injury of the hindlimb sensorimotor cortex but not by injury to the left cortex [14, 70]. **b** In contrast, naltrindole, the δ-opioid antagonist, inhibits the effects of the left-sided injury but not those of the right-sided lesion

Administration of opioid peptides mimics the effects of a unilateral brain lesion by inducing HL-PA with left or right hindlimb flexion in rats with intact brain (Fig. 3a) [14, 93–96]. These findings suggest that the endogenous opioid system differentially to regulates the effects of the left and right brain lesions on the hindlimb contralesional postural and sensorimotor deficits. This hypothesis was tested with general and selective antagonists of opioid receptors [70] (Fig. 4). The antagonists blocked formation of HL-PA induced by the UBI, and interfered with the brain injury-induced changes in contralesional hindlimb withdrawal reflexes. Strikingly the effects of the antagonists depended on the side of brain lesion. The µ-antagonist β-funaltrexamine and κ-antagonist nor-binaltorphimine (nor-BNI) reduced the asymmetry after the right but not left UBI (Fig. 4a). In contrast, the δ-antagonist naltrindole inhibited HL-PA induced by the left but not right side brain injury (Fig. 4b). The side-specific actions of the antagonists are in agreement with the effects of opioid agonists. The left hindlimb flexion, that was inhibited by the µ- and κ-antagonists, was induced by the preferential endogenous µ-/δ-agonist Met-enkephalin and κ-agonists U50,488H, bremazocine and dynorphin [70, 93, 94]. Naltrindole, a δ-antagonist blocked formation of the right limb flexion in rats with the left-side UBI that was consistent with the effects of δ-antagonist Leu-enkephalin that caused the right limb to flex [93, 94]. The well-matched antagonist and agonist effects suggest that the right side UBI-induced formation of the left flexion is mediated by the µ- and κ-receptors (Figs. 3, 4a). In contrast, development of the HL-PA with right flexion induced by the left UBI is mediated by δ-receptor. Thus, the neural circuits that are functionally mirror-symmetric and control activities of the left and right hindlimb muscles, are likely regulated by the left- and right-side-specific opioid mechanisms. The opioid peptides could differentially target the left and right counterparts of these circuits, and in this way control the left–right balance in their functional performance. This bipartite mechanism may be based on lateralization of neuropeptides and their receptors, and operate locally in the spinal cord, or at the system level by controlling neural projections from the brain sensorimotor areas to contralateral motoneurons at several sites.

## The long-range left–right side-specific neuropeptide signaling: enkephalins as the triggers

An intriguing question is whether the bipartite side-specific opioid mechanism is short-acting and operates locally (e.g., within the lumbar spinal cord), or whether it integrates activities of neural circuits at several levels of the neuraxis into the co-regulated side-specific networks. In the latter “broadcasting mechanism”, the opioid peptides may spread for a long-distance through volume transmission in the cerebrospinal fluid [106], or through the blood as neurohormones to coordinate the left–right-sided activities of the nearby and distant areas of the central nervous system.

**Fig. 5:**
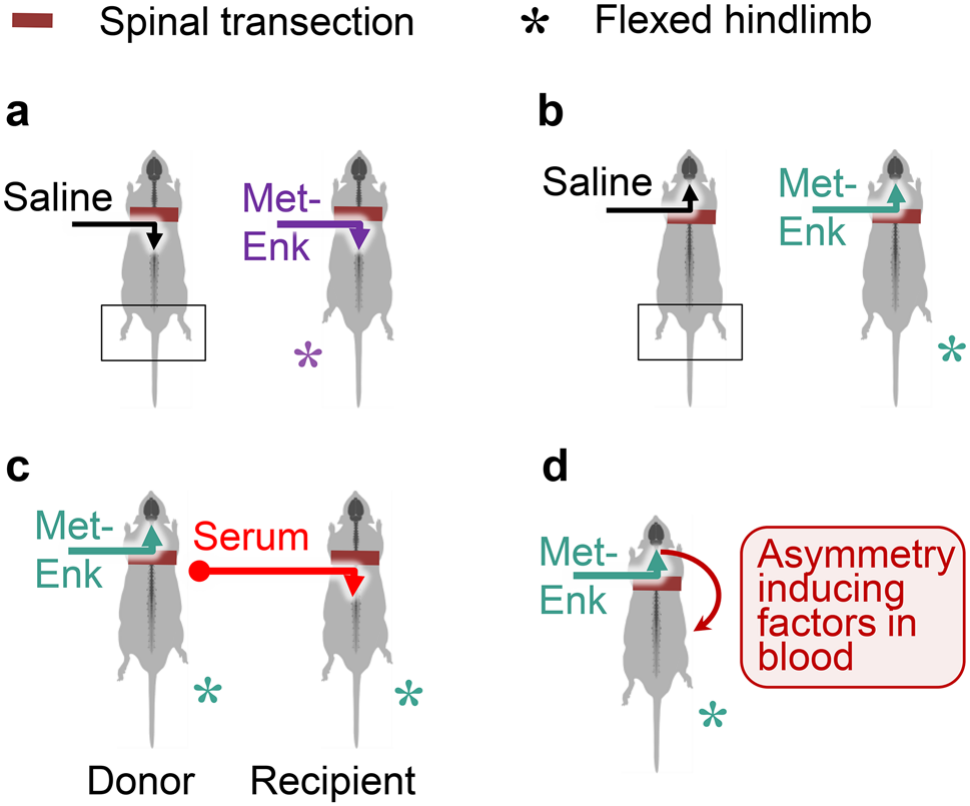
The enkephalin-evoked side-specific humoral signaling from the brain to the spinal cord. **a** Met-enkephalin (Met-Enk) administered intrathecally into the caudal part of completely transected spinal cord of rats with intact brains induced HL-PA with left hindlimb flexion [94, 95]. **b** HL-PA also developed after injection of this peptide into the rostral part of the transected lumbar spinal cord of rats with intact brains [95]. However, the flexion side was reversed; the right hindlimb was flexed. **c** Serum collected from animals given Met-enkephalin into the rostral spinal part (donors) produced HL-PA in the recipient rats. Serum was administered to the recipients intrathecally into the caudal portion of the transected lumbar spinal cord. The hindlimb was flexed on the same side (i.e., right side) in the donor and recipient animals [95]. Control serum produced no effects. **d** Model for humoral left–right side-specific signaling elicited by Met-enkephalin. Enkephalins could stimulate the release of peptide molecules—postural asymmetry inducing factors that convey the left–right side-specific message through the humoral pathway from the brain or endocrine glands to the lumbar spinal cord. Biochemical analysis demonstrated that these factors are short peptides that differ from Met- and Leu-enkephalins, endorphins, neoendorphins, and dynorphin (1–13) [95]

Evidence for the long-range left–right side-specific neuropeptide signaling came from analysis of the effects of the enkephalin opioid peptides on formation of HL-PA [95]. Intrathecal administration of Met-enkephalin into the caudal portion of the transected lumbar spinal cord induced HL-PA with the flexion of the left hindlimb [94–96] (Fig. 5a). Surprisingly after injection of this peptide into the rostral spinal part of the transected spinal cord, animals also developed the asymmetry [95]. But instead of the left limb, the right limb was flexed (Fig. 5b). The inversion in the flexion side was also observed for Leu-enkephalin. In opposite to Met-enkephalin, this peptide produced the right hindlimb flexion when it was administered into the caudal part of the transected spinal cord, while the left limb was affected after its injection above the transection level.

After rostral administration, enkephalins could penetrate into the blood, or stimulate the release of the postural asymmetry inducing factors from the brain or endocrine glands innervated by the neurons located above the section level, into the circulation. Then enkephalins or the postural asymmetry inducing factors may be transported by the blood to the lumbar spinal domains or peripheral endings of afferent neurons, where they induce postural asymmetry. To test this hypothesis, serum was taken from donor rats that received the rostral Met-enkephalin injection, and then the serum extract was intrathecally administered into the caudal part of the transected spinal cord in the recipient animals (Fig. 5c) [95]. Serum from donor animals treated with Met-enkephalin, produced HL-PA with the right hindlimb flexion in the recipients. Thus, the flexion was on the same side as it was in the donor rats. Control serum from animals treated with saline did not induce HL-PA. Biochemical analysis demonstrated that molecular factors in serum that induced the right-side flexion were thermostable while their activity was abolished by pre-incubation with proteolytic enzymes. These factors were eluted in a single fraction after liquid chromatography on Ultrasphere C-8 column and their retention time differed from those of Met- and Leu-enkephalins, α- and γ-endorphins, α- and β-neoendorphins and dynorphin (1–13) [95]. Thus enkephalins might stimulate a release of peptide molecules, possibly neurohormones, which convey the left–right side-specific message from the brain or endocrine glands to the lumbar spinal cord through the humoral pathway (Fig. 5d). In view of these findings, we will next discuss the asymmetry of the neuroendocrine system, the effects of brain lesions on this system, and humoral transmission of side-specific signals from the injured brain to the spinal cord by neurohormones.

## Asymmetric control of the neuroendocrine system

Several lines of evidence demonstrate that the neural control of the hypothalamic–pituitary–adrenal and hypothalamic–pituitary–gonadal axes is left–right hemisphere specific [107–114]. The right-sided injuries to the brain or spinal nerves compared to those on the left side generally produced stronger effects on the neuroendocrine and neuropeptide systems, including hormonal levels in the peripheral circulation [84, 108, 110, 115]. Cerebral laterality was described as an important factor in the regulation of stress responses, with the prominent role of the right hemisphere in control of the hypothalamic–pituitary–adrenal axis [110]. Analysis of the left- and right-sided infarctions revealed that the increase in morning cortisol level is under excitatory control of the right hemisphere. Stress and stress-induced pain produced lateralized neuropeptide and neuroendocrine responses in the brain and spinal cord [107, 113, 116, 117]. The prefrontal cortex in the right hemisphere was involved in responses of the hypothalamic–pituitary–adrenal axis to stress and regulation of the neuroendocrine system [113]. Stress and stress-induced functional pain are processed in the right amygdala where they are controlled by the lateralized κ-opioid system [117, 118]. Similarly, unilateral nerve and body injuries elicit functional and molecular neuropeptide and neurohormonal responses that are more pronounced on the right side in the brain and spinal cord [84, 107, 108, 115, 119].

Gonadotropin-releasing hormone is asymmetrically distributed between the left and right hypothalamus [107]. Unilateral castration by removal of the left testis resulted in substantial reduction of this hormone in the right hypothalamus but did not affect its content on the left side. The right-side castration produced no effects. In animals subjected to cold stress, gonadotropin-releasing hormone was affected again in the right hypothalamus while its content on the left side remained unchanged.

Notably, the hypothalamic–pituitary axis exhibits asymmetric functional patterns. Basal and corticotropin-releasing hormone-induced secretion of Arg-vasopressin and adrenocorticotropic hormone by the pituitary gland is lateralized to the right petrosal sinus [120]. A functional role of this asymmetric secretion is still to be investigated. Tonus of the hypothalamic–pituitary–adrenal axis is also associated with microstructural asymmetry in the hippocampus [111]. Left–right differences in diffusivity are linked to the cortisol levels that suggests an asymmetric role of this area in the neuroendocrine regulation.

The side-specific control of the neuroendocrine system including peripheral endocrine processes could be performed either by neural circuits with unusual, asymmetric organization, or by the left–right side-specific neuroendocrine regulators acting through paracrine or humoral mechanisms. A non-neural side-specific signaling from the brain to the paired endocrine glands or to the left and right spinal cord has been suggested in early studies [107, 121, 122]. This pathway has been recently supported by experimental evidence [15] and will be discussed in the next sections.

## The neuroendocrine effects of brain injury

TBI and stroke cause dysfunction of the hypothalamic–pituitary system and hypopituitarism with a prevalence of approximately 30% [123–129]. In most individuals a single pituitary axis is affected, while damage of multiple axes is less frequent. The most sensitive axes are growth hormone and gonadotropins while deficits of the adrenocorticotropic hormone and thyroid-stimulating hormone axes are less common. Hypopituitarism persists for a long time and aggravates the functional outcome.

TBI and stroke can affect the hypothalamus and pituitary gland directly or through compression of surrounding tissues [130, 131]. Cortical projections to the hypothalamus may possibly mediate the effects of focal brain injury on secretion of pituitary hormones [132]. Secondary injuries that include hypoxic insult, neurotransmitter-mediated excitotoxicity, axonal injury, inflammation, and autoimmunity could also lead to pituitary dysfunction [131]. The resulting cell death, cerebral edema and increased intracranial pressure can exacerbate brain damage. Inflammation in the hypothalamus and pituitary gland could evoke autoimmune processes and generation of antibodies against hypothalamic and pituitary antigens that lead to impairment of the neuroendocrine system.

Corticotropin-releasing factor, the hypothalamic neurohormone probably propagates the effects of neurovascular injury. Ischemic stroke activates the hypothalamus–pituitary–adrenal axis [133], while ablation of sensorimotor cortex elevates the level of circulating adrenocorticotropic hormone and induces morphological changes in the pituitary corticotrophs that produce adrenocorticotropic hormone and β-endorphin [134]. Expression of corticotropin-releasing factor in several brain areas including the hypothalamus is elevated following cerebral ischemia [135, 136]. In the acute phase of ischemic stroke, corticotropin-releasing factor and anti-inflammatory signals are possibly beneficial, while in the chronic phase, they contribute to neurodegeneration, toxicity and cell death [137]. Abnormal thyroid hormone metabolism is another sequelae of ischemic stroke that is characterized by the low levels of triiodothyronine in serum while concentrations of thyroid-stimulating hormone remain normal [138].

In summary, TBI and stroke have been recognized as prominent causes of hypopituitarism. This issue has been reviewed in several recent articles [125–127, 137, 139, 140]. The rest of this Review is focused on the left–right side-specific neuroendocrine signaling (Fig. 1b) that may be a part of general neuroendocrine responses to TBI and stroke, and mediate the effects of these lesions on postural and motor deficits.

## The neuroendocrine “cross association” phenomenon

### The left–right side-specific humoral signaling mediates the effects of unilateral brain lesion

**Fig. 6:**
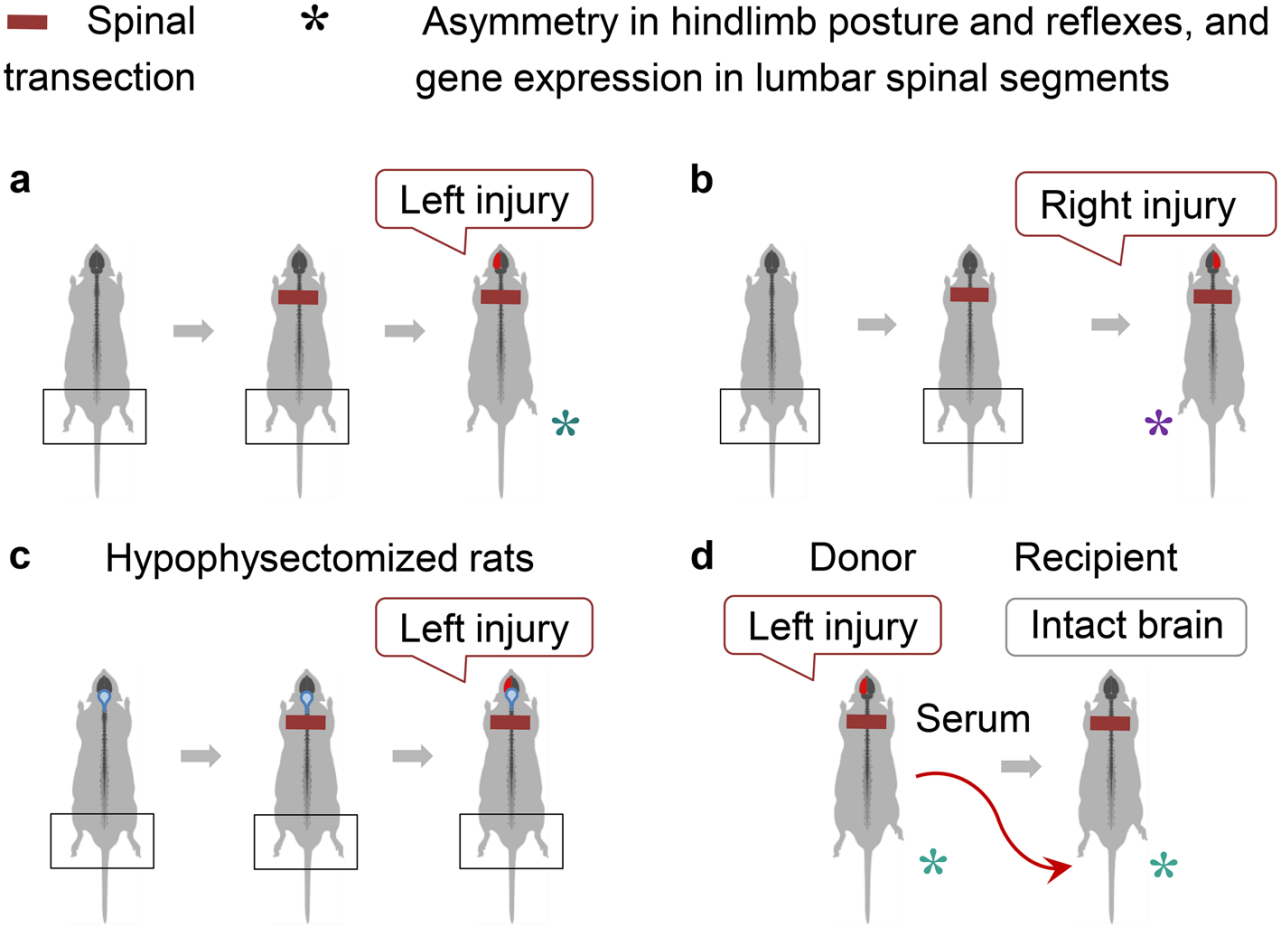
The left–right side-specific humoral signaling mediates the asymmetric effects of brain injury on hindlimb posture and reflexes. The neuroendocrine side-specific signaling was discovered in animals with the descending neural tracts that were disabled by complete transection of the spinal cord [15]. Unilateral ablation injury of the hindlimb sensorimotor cortex was performed after the spinal cord was transected. HL-PA, asymmetry in withdrawal reflexes of flexors and extensors of the left and right hindlimbs, and expression of neuroplasticity-related and neuropeptide genes in the lumbar spinal cord were analyzed as readouts. **a**, **b** The left-sided brain injury performed in rats after complete transection of the spinal cord induced HL-PA with right flexion while left flexion was developed after the right-sided brain injury. **c** Brain injury did not induce the asymmetry in hypophysectomized rats with completely transected spinal cords. **d** Intravenous administration of serum from rats with the left-sided brain injury (donor animals) induced HL-PA with right flexion in rats with intact brain and completely transected spinal cord (recipient animals)

**Fig. 7:**
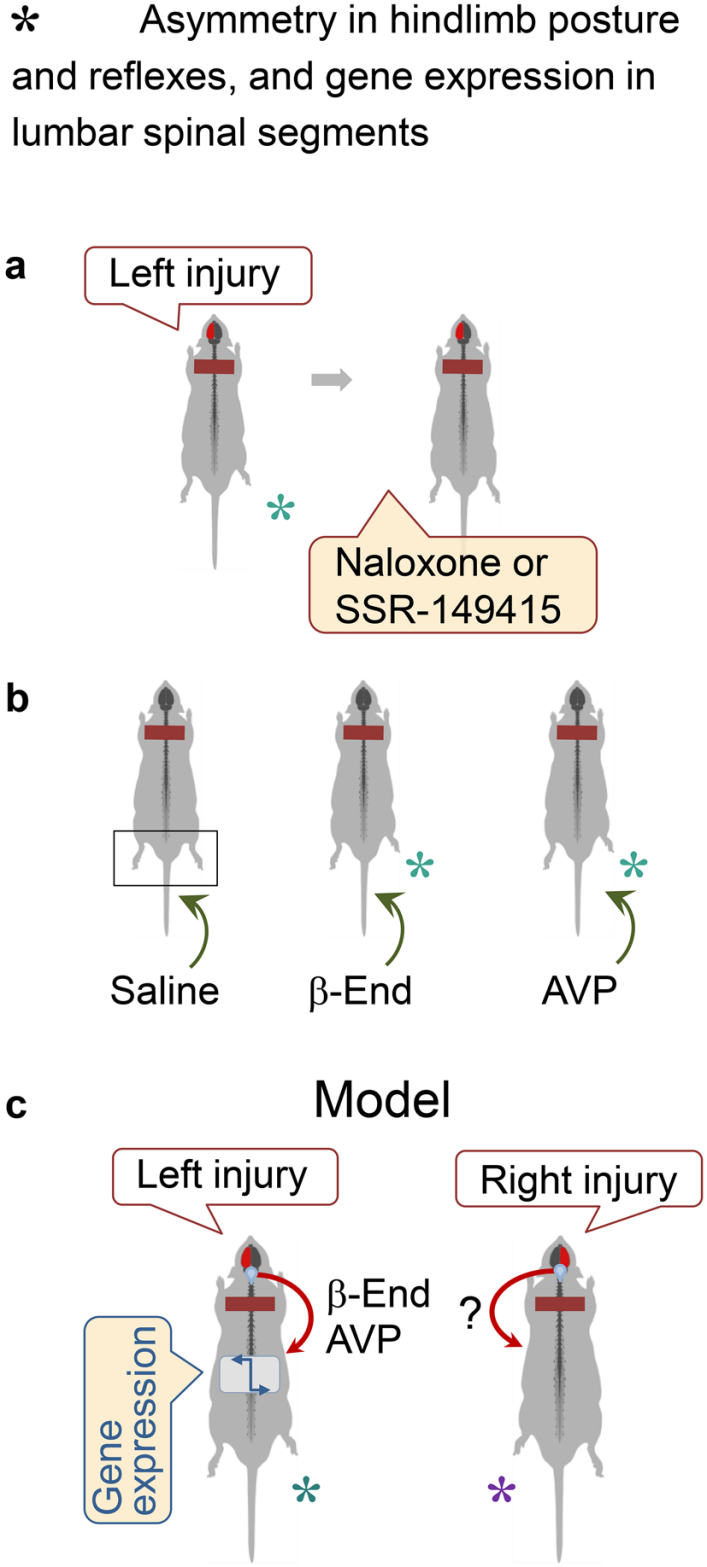
Pituitary neurohormones β-endorphin and Arg-vasopressin mediate the effects of the left-side brain injury. Model for the left–right side-specific neuroendocrine signaling from the injured brain. **a** Naloxone, the general opioid antagonist, and SSR-149415, the antagonist of the Arg-vasopressin V1B receptor expressed in the pituitary gland, inhibited the HL-PA that was induced in rats with transected spinal cord by injury to the left cortex. **b** Induction of the HL-PA with right hindlimb flexion by β-endorphin (β-End) or Arg-vasopressin (AVP) that were administered intravenously to rats with intact brain and completely transected spinal cord. **c** Model for the left–right side-specific humoral mechanism that mediates asymmetric effects of unilateral brain lesions on hindlimb posture and motor functions. The unilateral brain injury stimulates the release of pituitary hormones into the blood. These hormones activate their receptors that are lateralized in the spinal neural circuits or peripheral endings of afferent neurons and produce the contralateral hindlimb responses. The effects of the left-sided brain injury are mediated by Arg-vasopressin and β-endorphin. The hormones that are involved in response to the right-sided brain lesion have not been identified, although dynorphin and Met-enkephalin that induce flexion of the left hindlimb [14, 70] are the candidates

The left–right side-specific opioid mechanism [14, 70, 84, 93–96] besides control of local processes in the spinal cord, could mediate long-range signaling from the injured brain hemisphere to the contralateral extremities through the humoral pathway and thus bypass the descending neural tracts. This hypothesis was tested by analysis of the effects of UBI on the hindlimb posture and sensorimotor functions in animals with complete spinal cord transection (Figs. 6, 7) [15]. To reveal the endocrine signaling, the descending neural influences were disabled by complete transection of the spinal cord before the brain was injured. Thoracic transection was performed and then the hindlimb representation area of the sensorimotor cortex was ablated. Asymmetries of hindlimb posture, hindlimb withdrawal reflexes, and gene expression patterns in the lumbar spinal cord were studied as readouts of the brain injury effects.

In spite of complete transection of the spinal cord, the UBI induced HL-PA (Fig. 6a, b). Remarkably, the contralesional hindlimbs were affected. The left hindlimb flexion was induced by the right-sided brain injury, while lesion of the left hemisphere resulted in flexion of the right hindlimb. Besides HL-PA, UBI produced asymmetry in withdrawal reflexes [15]. In rats with transected spinal cord, UBI differently affected withdrawal reflexes of the contra- and ipsilesional hindlimb muscles including the extensor digitorum longus, interosseous, and semitendinosus. The asymmetric effects can be developed due to the increased sensitivity of the afferent circuits and/or elevated excitability of efferent systems on the contra vs. ipsilesional side. Sham surgery produced no effects.

Furthermore, UBI performed after complete spinal transection produced molecular changes in the lumbar spinal segments [15]. Expression of the neuroplasticity-related, opioid and vasopressin genes was decreased on the contralesional vs. ipsilesional side in the UBI rats. Gene regulatory networks or coordination of gene–gene expression within and between the left and right halves of the lumbar spinal cord were strongly impaired by brain injury. The endogenous opioid system that is involved in the side-specific effects of brain injury was also affected. The levels of the proenkephalin marker Met-enkephalin-Arg-Phe and the prodynorphin-derived Dynorphin B and Leu-enkephalin-Arg were substantially elevated in the ipsilesional spinal cord in the UBI rats.

Analysis of HL-PA, withdrawal reflexes, gene expression and opioid peptides provides strong functional and molecular evidence for the left–right side-specific humoral signaling from the injured brain to the lumbar neural circuits that assures the development of contralateral responses [15].

### Pituitary neurohormones mediate the left–right side-specific effects of UBI

The left–right side-specific humoral signaling may operate through a release of pituitary hormones into the blood. This is supported by the findings that the hypophysectomized animals with a UBI do not develop HL-PA (Fig. 6c) while blood collected from rats with brain lesion contains hormonal substances that induce postural asymmetry in rats with intact brain (Fig. 6d) [15]. Remarkably, the recipient animals injected with the serum, developed HL-PA with flexion on the same side as that of the donor animals. The magnitude of the HL-PA was similar in UBI rats with intact or transected spinal cords.

The pituitary gland is the main source of β-endorphin, the most potent opioid peptide in the body. Its release is stimulated by the antidiuretic hormone Arg-vasopressin that acts through the V1B receptor expressed in pituitary corticotrophs producing proopiomelanocortin, a precursor to β-endorphin [141]. Analysis of pituitary neurohormones identified β-endorphin and Arg-vasopressin as humoral factors that induce response of the right hindlimb after left side brain injury [15]. Naloxone, a nonselective opioid antagonist, and SSR-149415, the selective antagonist of the pituitary vasopressin V1B receptor, blocked the asymmetry formation in animals with transected spinal cords and lesion of the left hemisphere (Fig. 7a) [15]. β-Endorphin and Arg-vasopressin injected into animals with intact brain evoked asymmetric motor responses with the right hindlimb flexion (Fig. 7b) that was on the same side as the flexion after the injury of the left cortex.

Thus several lines of evidence demonstrate that topographical information may be conveyed by molecular messengers circulating in the blood and converted into the left–right side-specific motor responses (Fig. 7c). In this phenomenon, the opioid peptide β-endorphin and the antidiuretic hormone Arg-vasopressin can serve as the side-specific humoral messengers that signal from the injured brain to the spinal neural circuits. These neurohormones could act in parallel through the pathways that independently evoke the asymmetric responses, or mediate the consequent steps in the same route that leads to formation of HL-PA. The vasopressin receptor V1B is mainly expressed in the anterior pituitary by corticotrophs producing proopiomelanocortin [141]. In one scenario Arg-vasopressin released from neurohypophysis may activate the V1B receptor on corticotropes in the anterior pituitary and stimulate secretion of the proopiomelanocortin-derived β-endorphin that then induces the HL-PA. In the second mechanism, Arg-vasopressin and β-endorphin could act through the complex of the vasopressin receptor V1B and μ-opioid receptor that integrates two signaling pathways [142] and thus produce synergistic asymmetric effects.

### Intergenerational effect of brain injury

The developing nervous system is sensitive to environmental influences that could affect pregnant females. Several environmental effects are transmitted to the next generation and cause neuropsychiatric disorders (reviewed in [143]. Intergenerational transmission has been reported for stress, anxiety, cocaine use, and enhanced synaptic plasticity. By analogy, unilateral TBI and stroke in pregnant females may affect the developing neuroendocrine system and through the intergenerational mechanism cause neurological deficits in the offspring. This hypothesis has been tested with the HL-PA model [143]. The main finding was that the unilateral ablation injury of the hindlimb sensorimotor cortex in pregnant rats resulted in formation of HL-PA in the offspring. Remarkably, the transmitted effects depended on the side of brain injury. For example, after left UBI in the pregnant animals, the contralesional hindlimb in the progeny was affected. The asymmetry persisted after complete spinal transection in the offspring. Right UBI also caused the offspring to develop HL-PA; however, the asymmetry was cryptic and expressed only after spinalization. The HL-PA may develop due to an impaired balance between the left and right neuroendocrine signaling in the pregnant dams with UBI and the offspring, which is encoded in spinal neurocircuits.

Intergenerational transmission of neurological deficits can model several features of unilateral cerebral palsy [16]. This most common motor disability in childhood is characterized by asymmetry in posture, coordination, balance, and muscle tone. In most cases its causality has not been established. It was hypothesized that small cryptic lateralized brain lesions in pregnant women activate the left or right side counterparts of the bipartite neuroendocrine system, and this disbalance asymmetrically affects the developing CNS and leads to asymmetric neurological deficits in the offspring [16]. To note, due to complexity of the model, the intergenerational findings should be interpreted with caution, as they require further elaboration including assessment of the maternal and offspring's neuroendocrine system and neurological status and spinal neuroplasticity in the progeny.

## Conclusions and perspectives

In this review, we have discussed the recently discovered left–right side-specific neuroendocrine signaling, or the neuroendocrine “cross association” phenomenon. This signaling, in addition to descending neural tracts, mediates the asymmetric effects of unilateral brain lesions on posture and spinal reflexes, and thus determine whether the contralesional or ipsilesional side is affected (Figs. 1, 7b, c) [15]. The endocrine signals are released by the pituitary gland and can operate through the lateralized receptors in the lumbar spinal cord [70, 84] or on the peripheral terminals of sensory neurons. The humoral mechanism targets the expression of neuroplasticity-related genes and gene regulatory networks in the lumbar spinal segments. Changes in gene expression may tonically contribute to pathological spinal plasticity and consequently to persistent functional deficits induced by brain lesions.

Discovery of a novel phenomenon generally consists of two stages. The discovery phase is the acquisition of primary evidence, and is often accomplished with simple observational techniques as elegantly described by Hans Selye in his “In Vivo” lectures [144]. The second stage is the analysis of the mechanisms, biological role and clinical significance of the phenomenon by advanced methods. Evidence for the left–right side-specific neuroendocrine signaling came in substantial part from analysis of HL-PA that is a proxy for postural effects of brain lesions and quantitative, reproducible and technically simple animal model. This signaling has been discovered recently [15] and is currently entering the second phase. A number of conceptual and mechanistic questions are yet to be addressed. It would be worthwhile to identify sensorimotor and postural functions which of the impairments caused by unilateral brain lesions are controlled by the humoral signaling, and to determine a proportion of neurological deficits that are accounted for by the left–right side-specific neuroendocrine signaling vs. those mediated by neural pathways. It is essential to ascertain if the activities of the neural and endocrine pathways are additive, synergistic, or antagonistic with respect to each other. Pathways from the injured cortex to the hypothalamus, the neurohormones that mediate the effects of the right side injury, the central and peripheral targets for the left- and right-side-specific neuroendocrine messengers, and the neurophysiological mechanisms of the asymmetry formation should be also identified and investigated.

It has not yet been analyzed if the left–right side-specific neuroendocrine mechanism mediates the effects of brain lesions on the forelimb posture and functions. No changes in the forelimbs in the postural asymmetry model were evident after an ablation of the hindlimb sensorimotor cortex, suggesting an anatomical specificity of injury effects [13]. It is important to examine if neurohormones that selectively affect the left or right hindlimbs would also target forelimb functions, and if the fore and hindlimbs would respond to neurohormonal stimulation on the same or opposite side.

The side-specific neuroendocrine signaling is blocked by the opioid and vasopressin antagonists [14, 15, 70], and contralateral effects of brain lesions are reversed to the symmetric level. These effects suggest that the neural circuits that control contraction of the left and right hindlimb muscles, are intact in animals with brain trauma but rewired and fail to operate normally under tonic actions of pathogenic neurohormones. The findings with neurohormonal antagonists corroborate previous experimental and clinical observations that demonstrated that the general opioid antagonists naloxone and naltrexone reverse asymmetric neurological deficits secondary to unilateral cerebral ischemia [145–153], and lessen spasticity in patients with multiple sclerosis [154]. It is important to identify the clinical and pathophysiological features of asymmetric postural deficits, which are controlled by neurohormones, and the targeting of which by neurohormonal antagonists may promote functional recovery in TBI and stroke patients. Characterization of asymmetric sensorimotor deficits transmitted by the neuroendocrine “cross association” mechanism vs. features mediated by the neural “cross association” pathways is essential for the understanding of TBI and stroke.

## Data Availability

This article is a literature review and does not contain any new data.

## Abbreviations

HL-PA: Hindlimb postural asymmetry
TBI: Traumatic brain injury
UBI: A unilateral ablation brain injury
U50,488H: Trans-3,4-Dichloro-N-methyl-N-[2-(1-pyrrolidinyl)cyclohexyl]-benzeneacetam
